# Endometriosis Causing Large Bowel Obstruction: A Case Report

**DOI:** 10.7759/cureus.37025

**Published:** 2023-04-02

**Authors:** Mohamed I Ragab, Amany M Altabba, Saud Hilmi, Khalid E Attia, Ahmed I Elnogoomi

**Affiliations:** 1 General Surgery, Al Kuwait Hospital, Sharjah, ARE; 2 Medicine, Al Qassimi Hospital, Sharjah, ARE; 3 Radiology, Al Kuwait Hospital, Sharjah, ARE; 4 Surgery, Cairo University Hospital, Cairo, EGY

**Keywords:** acute large bowel obstruction, rectosigmoid endometriosis, rectosigmoid mass, bowel endometriosis, primary anastomosis

## Abstract

Endometriosis is defined as the presence of endometrial glands and stroma in ectopic locations, with extrapelvic sites being less commonly affected. Only a few cases of colonic endometriosis causing acute bowel obstruction have been reported in the literature, in which resection and primary anastomosis were the treatments performed. We present the case of a 40-year-old female who presented with signs and symptoms of acute large bowel obstruction thought to be due to malignancy; however, further evaluation confirmed the diagnosis of rectosigmoid endometriosis. The management plan was an immediate laparotomy with rectosigmoid resection and primary anastomosis.

## Introduction

Endometriosis is classically defined as the presence of endometrial glands and stroma in ectopic locations, primarily the pelvic peritoneum, ovaries, and rectovaginal septum [1]. Although less frequent, other tissues such as the pleura, pericardium, small and large intestines, and diaphragm may also be affected [2]. Symptoms of endometriosis vary depending on the location, with dysmenorrhea, dyspareunia, chronic pelvic pain, irregular uterine bleeding, and infertility being the most commonly observed symptoms [1]. One of the rare symptoms of endometriosis is large bowel obstruction (LBO). However, it is to be noted that this occurrence is infrequent due to the large intraluminal diameter of the intestine [3].

In females of reproductive age, the prevalence of endometriosis ranges from 2% to 22%, depending on the populations studied and the diagnostic criteria employed [4]. The incidence of endometriosis is higher in females with dysmenorrhea, ranging from 40% to 60%, than in females with subfertility, ranging from 20% to 30% [4].

Although the pathophysiology of endometriosis is not fully understood, retrograde menstruation is the most widely recognized explanation for its cause [1]. This occurs when endometrial tissue and cells are transplanted retrogradely and attach to peritoneal surfaces, establish a blood supply, and invade nearby structures [4]. Other contributing factors may include iatrogenic implantations and genetic susceptibility [1].

In this report, we present a case of acute LBO due to rectosigmoid endometriosis. Surprisingly, the acute LBO was the first manifestation of the patient’s endometriosis, with no previous gynecological symptoms.

## Case presentation

A 40-year-old nulliparous female presented to the Emergency Department (ED) with abdominal pain and spurious diarrhea for the last five days. She also had multiple episodes of vomiting and was intolerant to oral intake, but she was still able to pass stool and flatus at the time. During the same week, she had presented to the same ED twice with the same complaints but unfortunately was misdiagnosed with a urinary tract infection as she kept improving on symptomatic treatment during her stay in the ED. She had regular menstrual cycles of 29 days with four to five days of bleeding on average. She denied dysmenorrhea, oligomenorrhea, menorrhagia, and dyspareunia. She also denied any bowel changes in the prior months and during her menses. There was no history of recent weight loss. She is a non-smoker, and her past medical history and surgical history were unremarkable.

On examination, she was generally well and comfortable but looked pale. She was vitally stable, with a temperature of 37 °C, heart rate of 84 beats per minute, respiratory rate of 18 breaths per minute, blood pressure of 132/81 mmHg, and oxygen saturation of 100% on room air. Abdominal examination revealed a soft distended abdomen, as well as mild tenderness in the suprapubic region and left iliac fossa, with hyperactive bowel sounds. Laboratory tests showed C-reactive protein in the normal range (6 mg/L) and a negative beta-human chorionic gonadotropin test. Other labs including hemoglobin (12.10 g/dL), white blood cell count (10.91 × 10^9^/L), creatinine (49 µmol/L), serum albumin (32.40 g/L), and electrolytes were within the normal range.

**Figure 1:**
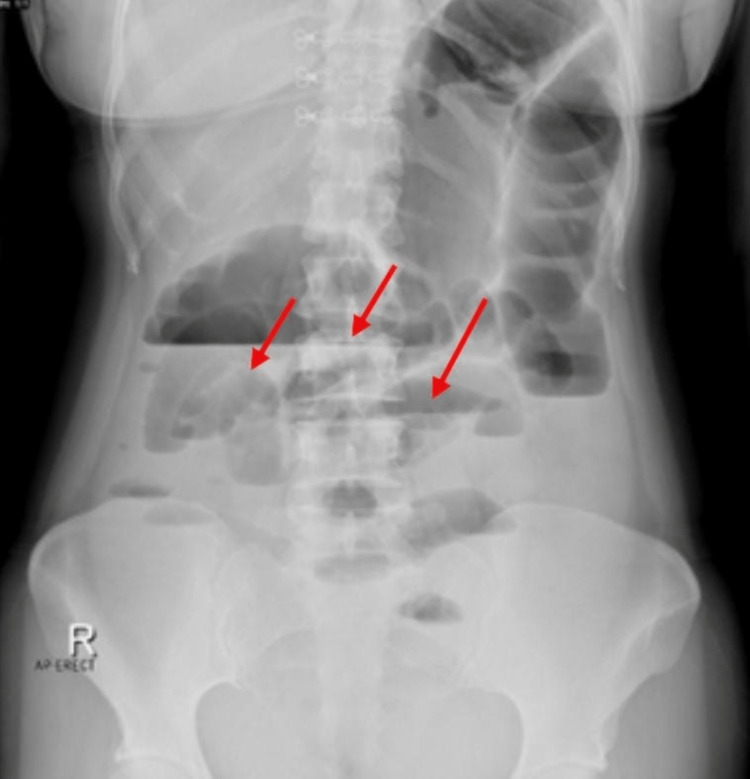
Abdominal X-ray showing dilatation of large and small bowel loops (red arrows).

**Figure 2:**
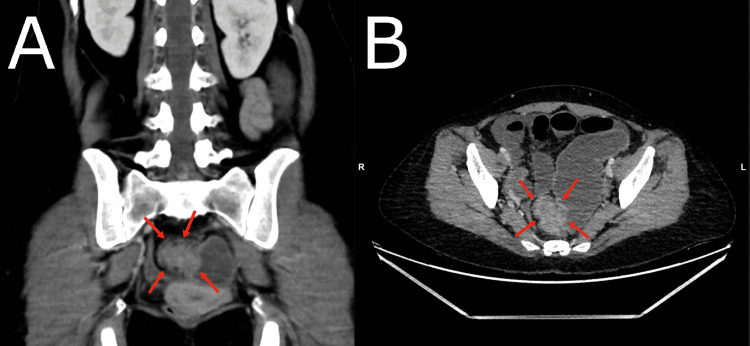
Abdominal CT scan with intravenous contrast showing a lobulated obstructing and enhancing mass lesion at the rectosigmoid junction (red arrows) in both coronal (A) and axial (B) sections. CT, computed tomography

**Figure 3:**
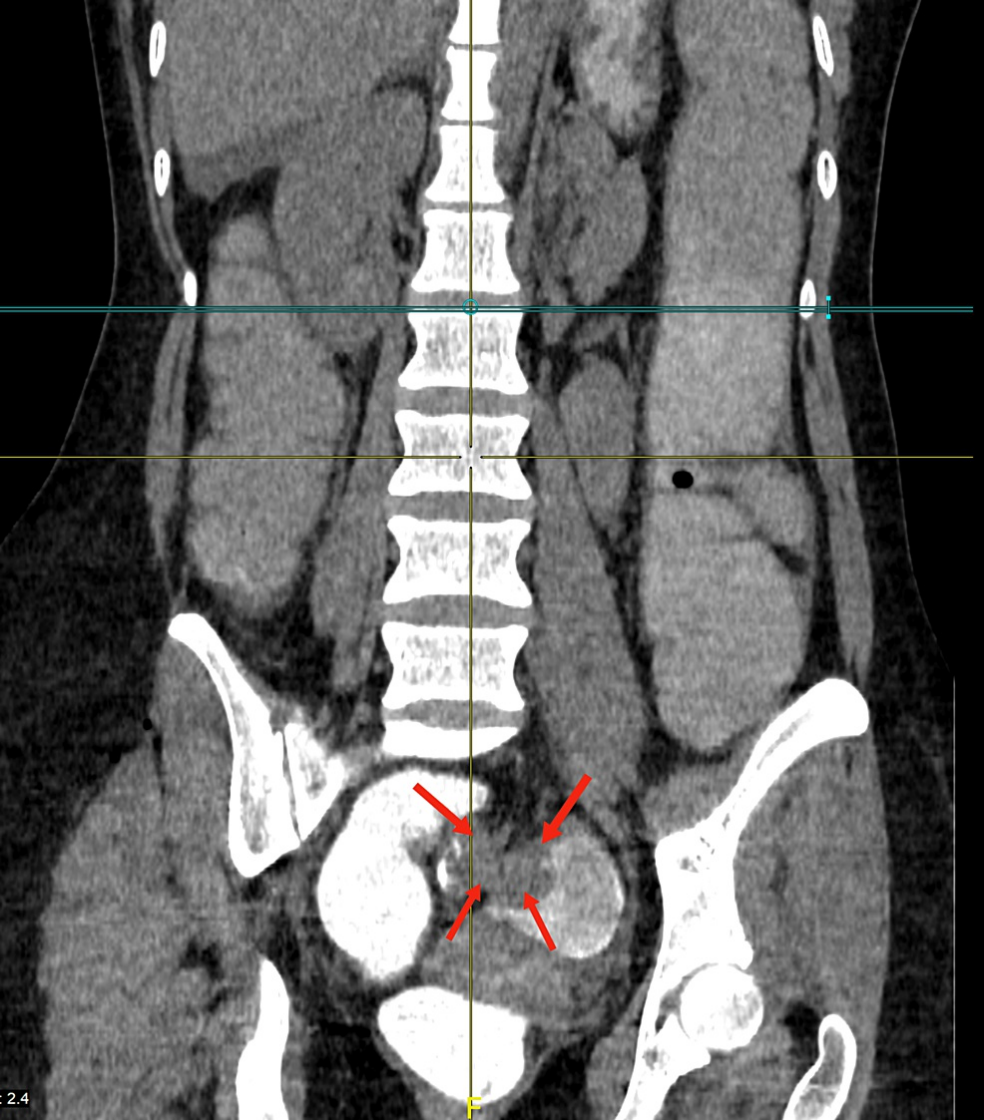
Abdominal CT scan with rectal contrast showing a filling rectum with a minimal trickling of contrast proximal to an obstructive mass (red arrows). CT, computed tomography

Abdominal X-ray showed distended bowel with multiple air-fluid levels, as can be seen in Figure 1. A computed tomography (CT) scan of the abdomen showed a lobulated mass in both the coronal and axial sections at the rectosigmoid junction 20 cm from the anal verge measuring approximately 4.5 cm x 3.6 cm, exhibiting enhancement after intravenous (IV) contrast injection, obliterating the lumen of the rectosigmoid, and causing an obstruction, as can be seen in Figure 2. A CT scan with rectal contrast showed filling of the rectum with the minimal trickling of contrast proximal to the lobulated obstructive mass at the rectosigmoid junction, as can be seen in Figure 3.

The patient was admitted after a surgical consultation suggesting a subacute bowel obstruction. Since the abdominal CT scan suggested an obstructive mass, a surgical approach was decided for the management of the patient. However, the patient refused any surgical intervention in the first two days of admission, as she was not in any severe condition and still passing stool and flatus. Per rectal examination revealed an empty rectum with no blood or mucus. A baseline carcinoembryonic antigen (CEA) tumor marker test was performed due to suspicion of malignancy, but the result was within the normal range (0.59 ng/mL). She was kept under direct observation until the third day of admission, where she developed obstipation with severe abdominal distention after which the patient agreed to surgical intervention. Colonoscopic stenting and decompression were attempted, but the guide wire failed to pass through the lumen. Thus, the procedure was abandoned, and the patient was prepared for urgent exploratory laparotomy.

Intraoperative findings included a rectosigmoid mass not fixed to the viscera or pelvis, obstructing the lumen completely at the rectosigmoid junction, with massive proximal distension. Exploration of the abdomen showed no evidence of peritoneal nodules, visceral metastases, or any other signs of endometriosis. Decompression was performed through an appendectomy stump, and an extended left hemicolectomy was conducted. The reason for choosing this procedure was due to compromised blood supply of the marginal arteries. During exploration, the segment of the colon proximal to the mass was found to be blue in color, indicating hypoperfusion that rendered the marginal arteries insufficient for primary anastomosis. However, the middle colic artery was found to be suitable for anastomosis, and therefore an extended left hemicolectomy was performed, along with primary anastomosis using a circular stapling gun.

**Figure 4:**
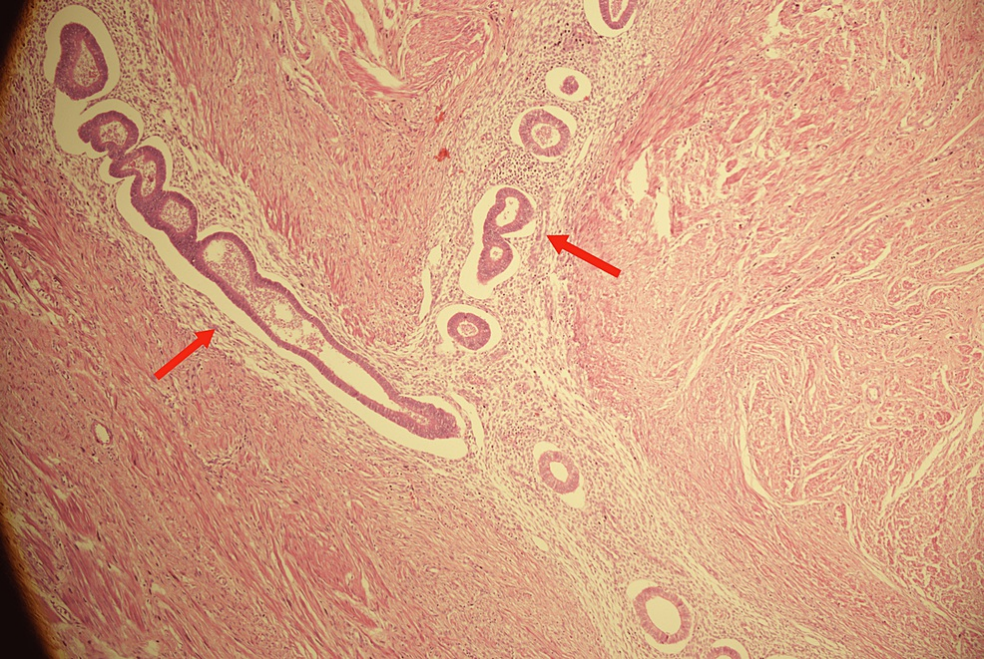
Endometrial glands and stroma in between colonic muscularis propria (red arrows) (H&E x200). H&E, hematoxylin and eosin

The histopathology report of the intramural mass with dimensions of 2.5 cm by 2 cm confirmed the diagnosis of colonic endometriosis by revealing islands of endometrial tissue formed of glands of variable size and shape and stroma in between (muscularis propria, serosa, and paraserosal mesenteric fat), as seen in Figure 4. No atypical cells could be detected.

The patient's recovery remained uneventful. She tolerated oral intake on the first postoperative day and got discharged on the eighth postoperative day. The patient was reviewed in the surgical outpatient clinic for two years, and she recovered uneventfully.

## Discussion

The presence of endometrial tissue in locations other than the uterus is known as endometriosis. The ectopic tissue could be mainly found in ovaries, rectovaginal septum, or even the pelvis [1]. To a lesser extent, endometriosis can also be found in the gastrointestinal tract in 3% to 34% of cases noted in the literature [5]. The clinical features of endometriosis are quite variable as it depends on the location and infiltration of the ectopic tissue. The most notable features are irregular and painful uterine bleeding, pelvic pain, dysmenorrhea, and dyschezia [1]. However, 70% of those who were diagnosed with the rare condition of gastrointestinal endometriosis presented with LBO along with the other features of endometriosis mentioned previously [6].

Endometriosis affecting the gastrointestinal tract was first noted in the literature back in the 1950s [7]. There are some theories regarding the pathogenesis of endometriosis such as retrograde menstruation and vascular dissemination; however, the pathogenesis still remains largely unascertained [8]. The mechanism by which this condition causes bowel obstruction is hypothesized to be due to the response of the smooth muscles in the bowel to the inflammation caused by the ectopic endometrial stroma. This, in turn, leads to metaplasia, hyperplasia, and fibrosis of the muscles, leading to obstruction [5].

The diagnosis of intestinal endometriosis can prove to be very challenging since there are numerous differentials that can present with similar symptoms, such as lymphoma, carcinoma, diverticulitis, and even irritable bowel disease. Imaging modalities such as pelvic or abdominal CT may only reveal the thickening of the intestinal wall and narrowing of the lumen. Although pelvic magnetic resonance imaging (MRI) and transvaginal ultrasonography (US) can also be used, they have limitations in identifying intestinal tissue deposits and adhesions caused by endometriosis [8]. Transvaginal US is the preferred modality since it is lower in cost and can detect the reduced flow of blood to the endometrioma using the Doppler technique, which is a characteristic finding of the disease [2].

The severity of the clinical presentation dictates the treatment of intestinal endometriosis. Medical management can be provided to patients with small deposits, which involves the use of nonsteroidal anti-inflammatory drugs (NSAIDs) along with combined oral contraceptives [8]. This approach aims to reduce the pain associated with the condition and provide time for an elective resection to be conducted later on. However, for larger deposits, surgical resection of the bowel is usually necessary [8].

Literature has reported four cases of sigmoid endometriosis that were treated surgically with primary anastomosis [8-11]. Three of these cases had either been already diagnosed with endometriosis or had symptoms of abdominal pain for at least six months prior to the presentation of LBO [8,10,11]. The fourth case primarily presented with acute bowel obstruction despite having no past medical history, similar to our patient [9]. All four cases showed significant improvement in the patient’s condition after the surgery and had no postoperative complications.

As in the case of this patient, she presented twice to the ED. Nevertheless, in both instances, she was misdiagnosed with a urinary tract infection. This might have been attributed to the vague symptoms she presented with and the improvement she exhibited on symptomatic treatment. However, it is worth noting that she denied any constipation but presented with diarrhea instead. This symptom should have raised suspicion of partial bowel obstruction, but, unfortunately, it was missed by the ED physician. As such, the clinician has to have a very high index of suspicion in order to reach this diagnosis. Moreover, radiographic imaging techniques and other procedures such as colonoscopy struggle with diagnosing intestinal endometriosis as the mucosal surface is usually unaffected which results in a normal biopsy in most cases. All of these limitations make laparoscopy the procedure of choice in order to confirm the diagnosis as long as the patient is stable and is not in complete obstruction [5].

In the case of our patient, she presented with symptoms of acute obstruction without any previous symptoms of endometriosis, which was quite interesting. Physical examination and initial investigations were not able to diagnose the patient as having endometriosis, which supports the difficulty of diagnosis in the case of intestinal endometriosis as mentioned in the literature [12].

## Conclusions

While endometriosis primarily targets the female reproductive system, it is also considered one of the rare causes of LBO. However, the condition presents a tough challenge for physicians to diagnose due to its pathophysiology, even with imaging modalities such as MRI. Therefore, all physicians should keep it in mind while putting together clues from the patient's presentation, including their medical history, current symptoms, laboratory findings, and imaging in order to diagnose and provide prompt treatment to the patient. This, in turn, will reduce the risk of complications and ensure a better quality of life for the patient.
